# The impact of serotonin transporter genotype on default network connectivity in children and adolescents with autism spectrum disorders^☆^

**DOI:** 10.1016/j.nicl.2012.10.008

**Published:** 2012-11-07

**Authors:** Jillian Lee Wiggins, Scott J. Peltier, Jirair K. Bedoyan, Melisa Carrasco, Robert C. Welsh, Donna M. Martin, Catherine Lord, Christopher S. Monk

**Affiliations:** a Department of Psychology, University of Michigan, 530 Church St., Ann Arbor, MI 48109, USA; b Functional MRI Laboratory, University of Michigan, 2360 Bonisteel Blvd., Ann Arbor, MI 48109, USA; c Department of Pediatrics, University of Michigan, 1150 W. Medical Center Dr., Ann Arbor, MI 48109, USA; d Neuroscience Program, University of Michigan, 530 Church St., Ann Arbor, MI 48109, USA; e Department of Radiology, University of Michigan, 2360 Bonisteel Blvd., Ann Arbor, MI 48109, USA; f Department of Psychiatry, University of Michigan, 4250 Plymouth Rd., Ann Arbor, MI 48109, USA; g Department of Human Genetics, University of Michigan, 1241 E. Catherine St., Ann Arbor, MI 48109, USA; h Department of Psychiatry, Weill Cornell Medical College, 21 Bloomingdale Road, White Plains, New York, NY 10605, USA; i Center for Human Growth and Development, University of Michigan, 300 N. Ingalls Building, 10th Floor, Ann Arbor, MI 48109, USA

**Keywords:** Functional MRI, Resting connectivity, Development, Serotonin transporter gene, Autism, Default network

## Abstract

Compared to healthy controls, individuals with autism spectrum disorders (ASD) have weaker posterior–anterior connectivity that strengthens less with age within the default network, a set of brain structures connected in the absence of a task and likely involved in social function. The serotonin transporter-linked polymorphic region (5-HTTLPR) genotypes that result in lowered serotonin transporter expression are associated with social impairment in ASD. Additionally, in healthy controls, low expressing 5-HTTLPR genotypes are associated with weaker default network connectivity. However, in ASD, the effect of 5-HTTLPR on the default network is unknown. We hypothesized that 5-HTTLPR's influence on posterior–anterior default network connectivity strength as well as on age-related changes in connectivity differs in the ASD group versus controls. Youth with ASD and healthy controls, ages 8–19, underwent a resting fMRI acquisition. Connectivity was calculated by correlating the posterior hub of the default network with all voxels. Triallelic genotype was assessed via PCR and Sanger sequencing. A genotype-by-diagnosis interaction significantly predicted posterior–anterior connectivity, such that low expressing genotypes (S/S, S/L_G_, L_G_/L_G_) were associated with stronger connectivity than high expressing genotypes (L_A_/L_A_, S/L_A_, L_A_/L_G_) in the ASD group, but the converse was true for controls. Also, youth with ASD and low expressing genotypes had greater age-related increases in connectivity values compared to those with high expressing genotypes and controls in either genotype group. Our findings suggest that the cascade of events from genetic variation to brain function differs in ASD. Also, low expressing genotypes may represent a subtype within ASD.

## Highlights

► Youth with autism and controls genotyped for 5-HTTLPR ► Posterior–anterior default network connectivity assessed with MRI during rest ► Stronger connectivity in low versus high expressing genotypes in autism ► Weaker connectivity in low versus high expressing genotypes in controls ► Largest age-related increases in connectivity in ASD with low expressing genotypes

## 1 Introduction

Autism spectrum disorders (ASD) are neurodevelopmental conditions characterized by social and communicative impairments and rigid repetitive behaviors. The prevalence of ASD has sharply increased in recent years and is currently 1 in 88 (CDC, 2012). Deciphering the complex etiology of ASD is thus a priority, and progress will likely involve examining the condition using multiple methodologies, including neuroimaging and molecular genetics.

As alterations in brain connectivity have been repeatedly implicated in ASD (Hughes, 2007), attention has been focused on identifying perturbations in fundamental, large-scale networks, such as the default network, that may contribute to ASD symptoms. In healthy adults, the default network (including the posterior cingulate, angular gyri, superior frontal gyri/Brodmann's area (BA) 10, and anterior cingulate/BA 10) is active and functionally connected in the absence of a demanding task (Raichle and Snyder, 2007). Functional connectivity reflects structural connectivity of the default network in healthy adults (Greicius et al., 2009). The default network contains posterior and anterior hubs (Buckner et al., 2008) that typically display strong long-range connectivity but are distinct from one another (Horovitz et al., 2009).

The primary purpose of the default network is a subject of debate. The default network may relate to basic central nervous system functions such as maintaining the balance of excitatory and inhibitory inputs or interpreting information from the environment (Raichle and Snyder, 2007). Alternatively, the primary purpose of the default network may be related to social cognition, including self-referential processes (Gusnard et al., 2001) and mentally projecting oneself into hypothetical situations (Buckner and Carroll, 2007).

Studies on adults with ASD (Cherkassky et al., 2006; Kennedy and Courchesne, 2008; Monk et al., 2009) as well as adolescents (Weng et al., 2010; Anderson et al., 2011; Wiggins et al., 2011) found weaker connectivity between the posterior and anterior default network compared to controls. Moreover, the weaker the posterior–anterior default network connectivity, the worse the social impairment in individuals with ASD (Monk et al., 2009; Weng et al., 2010).

A few studies have investigated the development of the default network. For healthy individuals, posterior–anterior connectivity is weaker during childhood and adolescence than during adulthood both functionally (Fair et al., 2008; Stevens et al., 2009; Wiggins et al., 2011) and structurally (Supekar et al., 2010). These studies indicate that connectivity of this network increases in strength over childhood and adolescence in healthy individuals. In contrast, youth with ASD have attenuated increases in posterior–anterior connectivity with age compared to controls (Wiggins et al., 2011).

Identifying the genetic factors that influence the default network in ASD is important to further elucidate the complex etiology of ASD. The serotonin transporter-linked polymorphic region variant (5-HTTLPR; Lesch et al., 1996) in the promoter region of the serotonin transporter gene (*SLC6A4*) is relevant to the default network in ASD. The S and L_G_ alleles of 5-HTTLPR are associated with decreased serotonin transporter expression relative to the L_A_ allele (A to G SNP in the L allele, rs25531; Hu et al., 2006). The low expressing alleles of 5-HTTLPR have been associated with worse social symptoms in ASD (Tordjman et al., 2001; Brune et al., 2006). In healthy adolescents, 5-HTTLPR is known to influence the default network: those with low expressing genotypes exhibit weaker posterior–anterior connectivity than adolescents with high expressing genotypes (Wiggins et al., 2012). Moreover, in healthy children and adolescents, 5-HTTLPR also impacts the development of default network connectivity such that youth with high expressing genotypes have greater age-related increases in posterior–anterior connectivity than those with low expressing genotypes (Wiggins et al., 2012). A previous study found that serotonin transporter binding in the anterior default network is decreased in individuals with autism (Nakamura et al., 2010). However, no study has yet examined how 5-HTTLPR affects default network connectivity or its development in individuals with ASD.

The present study addresses these two gaps in the literature on ASD: the role of 5-HTTLPR in default network connectivity and in the development of default network connectivity. This is accomplished by directly examining the influence of 5-HTTLPR variants on posterior–anterior default network connectivity as well on as age-related changes in connectivity in a sample of children and adolescents with ASD and controls. We hypothesized that the relationship between 5-HTTLPR genotype and posterior–anterior default network connectivity strength differs in the ASD group versus controls. Additionally, we hypothesized that the relationship between 5-HTTLPR and changes in connectivity across childhood and adolescence differs in the ASD group compared to controls.

## 2 Material and methods

### 2.1 Participants

Fifty-four children and adolescents with ASD and 66 healthy controls, aged 8.3 to 19.6 years, were included in this study (see Table 1 for participant characteristics). From a total of 105 participants with ASD and 82 controls recruited, 51 participants with ASD and 16 controls were excluded because of head movement exceeding 2.5 mm translation or 2.5° rotation, declining to complete the MRI scan due to discomfort, failure to return a saliva sample for genotyping, or technical problems with the MRI.

Controls were recruited through flyers posted at community organizations in the Ann Arbor, Michigan area. The University of Michigan Autism and Communication Disorders Center (UMACC) referred potential participants to our study and diagnosed participants with an ASD (autistic disorder, Asperger's syndrome, or pervasive developmental disorder — not otherwise specified) using the Autism Diagnostic Observation Schedule (ADOS; Lord et al., 2000), the Autism Diagnostic Interview—Revised (ADI-R; Lord et al., 1994), and clinical consensus (Lord et al., 2006). The University of Michigan Institutional Review Board approved the procedures. Participants over age 18 gave written informed consent; participants under age 18 gave written assent and their parents gave written informed consent. Cognitive functioning was evaluated for controls with the Peabody Picture Vocabulary Test (PPVT; Dunn and Dunn, 1997) and the Ravens Progressive Matrices (Raven, 1960); participants with ASD were given these measures or the Differential Ability Scales II — School Age (Elliott, 2005), the Stanford–Binet Intelligence Scales (Roid, 2003), the Wechsler Intelligence Scale for Children IV (Wechsler, 2003), or the Wechsler Abbreviated Scale of Intelligence (Wechsler, 1999). Participants with orthodontic braces, medical conditions contraindicated for MRI, or history of seizures or neurological disorders were excluded. Control participants were screened for psychological disorders with the Child Behavior Checklist (Achenbach and Edelbrock, 1981), Social Responsiveness Scale (Constantino et al., 2003), Social Communication Questionnaire (Rutter et al., 2003), Obsessive Compulsive Inventory — Revised (Foa et al., 2010), Child Depression Inventory (Kovacs, 1992), and Multidimensional Anxiety Scale for Children (March et al., 1997). All control participants scored below clinical cutoffs for affected status. Individuals with the low and high expressing genotypes did not differ in any of the symptom measures or cognitive functioning in either the ASD or control group (Inline Supplementary Table S1). Prior studies utilized portions of this dataset (Weng et al., 2010, 2011; Wiggins et al., 2011, 2012).

Controls were recruited through flyers posted at community organizations in the Ann Arbor, Michigan area. The University of Michigan Autism and Communication Disorders Center (UMACC) referred potential participants to our study and diagnosed participants with an ASD (autistic disorder, Asperger's syndrome, or pervasive developmental disorder — not otherwise specified) using the Autism Diagnostic Observation Schedule (ADOS; Lord et al., 2000), the Autism Diagnostic Interview—Revised (ADI-R; Lord et al., 1994), and clinical consensus (Lord et al., 2006). The University of Michigan Institutional Review Board approved the procedures. Participants over age 18 gave written informed consent; participants under age 18 gave written assent and their parents gave written informed consent. Cognitive functioning was evaluated for controls with the Peabody Picture Vocabulary Test (PPVT; Dunn and Dunn, 1997) and the Ravens Progressive Matrices (Raven, 1960); participants with ASD were given these measures or the Differential Ability Scales II — School Age (Elliott, 2005), the Stanford–Binet Intelligence Scales (Roid, 2003), the Wechsler Intelligence Scale for Children IV (Wechsler, 2003), or the Wechsler Abbreviated Scale of Intelligence (Wechsler, 1999). Participants with orthodontic braces, medical conditions contraindicated for MRI, or history of seizures or neurological disorders were excluded. Control participants were screened for psychological disorders with the Child Behavior Checklist (Achenbach and Edelbrock, 1981), Social Responsiveness Scale (Constantino et al., 2003), Social Communication Questionnaire (Rutter et al., 2003), Obsessive Compulsive Inventory — Revised (Foa et al., 2010), Child Depression Inventory (Kovacs, 1992), and Multidimensional Anxiety Scale for Children (March et al., 1997). All control participants scored below clinical cutoffs for affected status. Individuals with the low and high expressing genotypes did not differ in any of the symptom measures or cognitive functioning in either the ASD or control group (Inline Supplementary Table S1). Prior studies utilized portions of this dataset (Weng et al., 2010, 2011; Wiggins et al., 2011, 2012).

Inline Supplementary Table S1

**Table S1:** 

	Autism Spectrum Disorders Group								Control Group							
	Low Expressing Genotypes			Higher Expressing Genotypes					Low Expressing Genotypes			Higher Expressing Genotypes				
	S/S	S/LG	LG/LG	LA/LA	S/LA	LA/LG			S/S	S/LG	LG/LG	LA/LA	S/LA	LA/LG		
**Number of participants**	10	5	1	12	25	1			18	3	1	21	22	1		
**Total N**	16			38					22			44				
															**χ2**	**p**
**Gender (F:M)**	1:15			7:31					6:16			11:33			3.76 (df = 3)	0.29
**Handedness (L:R:ambidextrous)**	4:11:00			5:28:01					4:18:00			3:38:00			6.17 (df = 6)	0.41
**Caucasian**	94%			90%					64%			82%				
															F(1,116)*	**p**
**Age**	13.5 (2.78)			13.9 (2.72)					14.8 (2.35)			14.1 (3.26)			1.11	0.295
**Verbal CF**	115 (25.6)			111 (18.8)					113 (13.1)			115 (13.7)			0.81	0.370
**Nonverbal CF**	113 (17.6)			101 (21.9)					103 (11.6)			101 (13.0)			2.756 (df = 1,112)	0.100
							t(52)**	**p**							t(64)***	**p**
**SRS**	74.5 (12.2)			78.0 (11.1)			1.01 (df = 51)	0.318	43.6 (7.68)			42.5 (6.37)			.590 (df = 63)	0.557
**SCQ**	19.4 (7.67)			21.73 (6.85)			1.05 (df = 46)	0.299	2.95 (2.84)			3.36 (4.23)			.400 (df = 59)	0.691
**MASC**	42.3 (21.5)			45.2 (16.8)			.509 (df = 47)	0.613	34.8 (14.1)			31.4 (15.2)			.847 (df = 60)	0.400
**CDI**	7.13 (4.26)			8.47 (6.38)			.748 (df = 51)	0.458	5.86 (4.00)			4.47 (5.27)			1.094 (df = 63)	0.278
**CBCL Internal**	60.0 (18.0)			56.4 (21.3)			0.537	0.593	45.0 (9.43)			45.2 (10.7)			0.09	0.926
**CBCL External**	49.0 (15.6)			51.7 (20.9)			0.457	0.650	44.4 (7.33)			41.7 (10.4)			1.09	0.279
**CBCL Total**	57.4 (17.1)			58.1 (22.0)			0.110	0.913	44.2 (8.81)			42.4 (10.48)			0.67	0.504
**OCI-R**	18.0 (13.8)			18.9 (13.3)			.225 (df = 47)	0.823	10.1 (6.81)			10.1 (9.10)			0.004 (df = 59)	0.997

Note: Some participants were missing data. This is noted with altered df. *df = 1,116 unless otherwise specified. **df = 52 unless otherwise specified. ***df = 64 unless otherwise specified. CF = cognitive functioning. SRS = Social Responsiveness Scale. SCQ = Social Communication Questionnaire – Lifetime. MASC = Multidimensional Anxiety Scale for Children. CDI = Children’s Depression Inventory. CBCL = Child Behavior Checklist. OCI-R = Obsessive Compulsive Inventory – Revised; Likelihood ratio test used for chi-square analyses.

Inline Supplementary Table S1

Inline Supplementary Table S1 can be found online at http://dx.doi.org/10.1016/j.nicl.2012.10.008.

### 2.2 Genetic analyses

5-HTTLPR genotype was ascertained using previously published procedures (Wiggins et al., 2012). Participants donated saliva samples using the Oragene DNA kit (DNA Genotek; Kanata, Canada). PCR and agarose genotyping were used to determine S versus L allele. Sanger sequencing was utilized to determine the A to G single nucleotide polymorphism (SNP) in the L allele (rs25531; Hu et al., 2006) and to confirm PCR genotyping.

In autism, individuals with the low expressing genotype (S/S) have been shown to differ in neurochemical metabolism compared to L allele carriers in the anterior portion of the default network (Endo et al., 2010). As such, participants were put into two genotype groups: low expressing genotypes (S/S, S/L_G_, L_G_/L_G_) versus medium and high expressing genotypes (L_A_/L_A_, S/L_A_, L_A_/L_G_, hereafter referred to as "high expressing" genotypes). (The L_G_ allele is equivalent to the S allele in serotonin transporter expression level (Hu et al., 2006), so for the purposes of the analyses, the two alleles were grouped together.) This genotype grouping is consistent with a number of non-ASD studies that found recessive effects of the low expressing 5-HTTLPR alleles, often in adolescent populations (e.g., Cicchetti et al., 2007; Surguladze et al., 2008; Benjet et al., 2010). Nevertheless, we conducted additional analyses to examine whether our results still stood when the alleles were grouped differently (see Section 3.1.4 Alternative genotype groupings).

Hardy–Weinberg equilibrium was tested based on the insertion/deletion polymorphism. Genotype frequencies were in Hardy–Weinberg equilibrium for the ASD group (*χ*^2^ = 0.742, df = 1, *p* = 0.389), but there was a trend toward disequilibrium for the control group (*χ*^2^ = 3.74, df = 1, *p* = 0.053). When including only Caucasians for the control group, the trend disappeared (*χ*^2^ = 0.654, df = 1, *p* = 0.419). Because of this, additional analyses were performed to address the potential effects of multiple ancestries within the sample.

### 2.3 fMRI data acquisition

T_2_*-weighted blood oxygen level dependent (BOLD) images were acquired during a resting state scan, in which participants were instructed not to think of anything in particular and to let their minds wander while looking at a fixation cross. Over the 10-minute resting state scan, 300 images were acquired (Glover and Law, 2001; TR = 2000 ms, TE = 30 ms, flip angle = 90°, FOV = 22 cm, 64 × 64 matrix, 40 contiguous axial 3 mm slices). Slices were acquired parallel to the intercommissural (AC–PC) line. High-resolution 3D T1 axial overlay (TR = 8.9, TE = 1.8, flip angle = 15°, FOV = 26 cm, slice thickness = 1.4 mm, 124 slices; matrix = 256 × 160) and spoiled gradient (SPGR; flip angle = 15°, FOV = 26 cm, 1.4 mm slice thickness, 110 slices) anatomical images were also collected. Participants wore a pulse oximeter and abdominal pressure belt to record cardiac and respiratory rhythms, synchronized to the fMRI data, for subsequent physiological artifact correction. Further details on the acquisition parameters have been previously published (Weng et al., 2011; Wiggins et al., 2012). Prior to the MRI scan, participants practiced in a mock scanner to acclimate to the scanning environment.

### 2.4 fMRI data analysis

#### 2.4.1 Data preprocessing

The standard pre-processing procedure from the University of Michigan Functional MRI Center was applied to the fMRI data. This procedure includes removing outliers from the raw k-space data, reconstructing the k-space data to image space, applying a field map correction to reduce artifacts from susceptibility regions, and correcting for slice timing. RETROICOR was utilized to remove noise associated with cardiac and respiratory rhythms (Glover et al., 2000). To address potential effects of head motion, functional images were realigned to the 10th image. Details on these steps are available in multiple papers utilizing this pre-processing stream (e.g., Weng et al., 2011; Wiggins et al., 2012). The high-resolution T1 anatomical images were then co-registered to the functional images using the SPM5 Matlab toolbox (Wellcome Department of Neurology, London, UK; http://www.fil.ion.ucl.ac.uk). After removing variance associated with head motion (see Section 2.4.2 Addressing head motion), functional images were smoothed with an isotropic 8 mm full width at half maximum (FWHM) Gaussian kernel using SPM5. A low-pass filter of .08 Hz was applied as well to isolate the frequency band where default network activation has been found.

#### 2.4.2 Addressing head motion

Recent papers have emphasized the importance of addressing head motion, which can introduce spurious correlations in connectivity analyses (Power et al., 2012; Satterthwaite et al., 2012; Van Dijk et al., 2012). In addition to the standard realignment of images to correct for head motion, we took several steps to address the potential effects of head motion on our results. First, we excluded participants whose head motion exceeded 2.5 mm in the x, y, or z direction or 2.5° in the roll, pitch, or yaw directions.

Second, we removed variance associated with head motion by creating nuisance regressors from motion estimated in the x, y, z, roll, pitch, and yaw directions and retaining the residuals for processing.

Third, we calculated the mean motion for each person (i.e., mean absolute displacement of each volume as compared to the previous volume, calculated as square root ((x_*i* + 1_ − x_*i*_)^2^ + (y_*i* + 1_ − y_*i*_)^2^ + (z_*i* + 1_ − z_*i*_)^2^) for *i* = 1, …, 300 images), as in Van Dijk et al (2012). We then conducted a 2-way ANOVA with post-hoc contrasts to examine whether mean motion differed between the ASD and control groups and by genotype. We also performed a 3-way interaction analysis to examine whether the interaction of genotype-by-diagnosis-by-age significantly predicted mean motion.

Fourth, even though groups may not differ on overall mean motion, it is possible that the different motion distributions within groups could influence findings. Because of this, we repeated the analyses for our main hypotheses with a subsample of our participants matched on mean motion to examine whether the results persisted when the groups' motion distributions were equivalent (see Section 3.1.1 Matched head motion distributions). Several other studies have utilized matching to reduce the likelihood that the connectivity patterns they observed were an artifact of head motion (Fair et al., 2007, 2008; Dosenbach et al., 2010). Our matching procedure was as follows: first, we split participants into four groups: individuals with the low expressing genotypes (ASD and control) and individuals with the high expressing genotypes (ASD and control). Within each group, we binned participants by mean motion into .001 mm bins. Participants were removed randomly until the number of participants in each corresponding bin for the ASD and control groups were the same within both the low and high expressing genotypes.

#### 2.4.3 Connectivity images

**Fig. S1:**
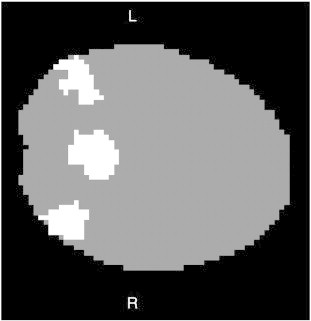
An example of the default network posterior hub identified through the self-organizing map algorithm. Following procedures described in Wiggins et al. (2011), the self-organizing map algorithm, a data-driven method, was applied to the data to organize voxels into networks. An experienced investigator blind to condition identified the network that contained the posterior hub (posterior cingulate and angular gyri/inferior parietal lobules) of the default network for each individual. The posterior hub was then used as an individualized reference to calculate default network connectivity for each participant. An example of the posterior hub from one individual is shown here. Data are from a single 64 × 64 slice in the transverse plane. White indicates that the voxel is a member of the posterior hub; gray indicates that the voxel does not belong in the posterior hub. The brain is masked for illustration purposes to highlight the posterior hub. At this point in the data-processing stream, brains are not yet normalized.

A self-organizing map algorithm was applied to the images to derive a data-driven reference from the posterior hub of the default network to calculate connectivity for each individual, as described in previous publications (Peltier et al., 2003; Wiggins et al., 2011, 2012). An example of a posterior hub identified for an individual using the self-organizing map algorithm is shown in Inline Supplementary Fig. S1. The advantage of using this data-driven method is that, unlike traditional a priori seed analyses, seed placement is not based on data from adult control brains. When using the self-organizing map algorithm, the default network reference, which is correlated with every other voxel in the brain to calculate connectivity, is not biased toward the control group but rather tailored for each individual.

A self-organizing map algorithm was applied to the images to derive a data-driven reference from the posterior hub of the default network to calculate connectivity for each individual, as described in previous publications (Peltier et al., 2003; Wiggins et al., 2011, 2012). An example of a posterior hub identified for an individual using the self-organizing map algorithm is shown in Inline Supplementary Fig. S1. The advantage of using this data-driven method is that, unlike traditional a priori seed analyses, seed placement is not based on data from adult control brains. When using the self-organizing map algorithm, the default network reference, which is correlated with every other voxel in the brain to calculate connectivity, is not biased toward the control group but rather tailored for each individual.

Inline Supplementary Fig. S1

Inline Supplementary Fig. S1 can be found online at http://dx.doi.org/10.1016/j.nicl.2012.10.008.

The connectivity images generated by this method were normalized to Montreal Neurological Image (MNI) space by estimating the transformation matrix for the SPGR image to SPM's template MNI image, and applying that transformation to the connectivity images. The end product is a normalized image for each subject that indicates, with a Z value at each voxel, how highly functionally connected (correlated) that voxel is to the posterior hub of the default network identified by the self-organizing map algorithm.

#### 2.4.4 Group-level analyses

The connectivity images were then entered into second-level analyses in SPM8 to test hypotheses at a group level. As a preliminary step, we first examined whether the ASD group and the control group exhibited default network connectivity by applying small volume corrections using masks covering the default network (posterior cingulate, precuneus, angular gyri, inferior parietal lobules, parahippocampal gyri, superior frontal gyri, anterior cingulate, BA 32, BA 10), from the Wake Forest Pickatlas (Maldjian et al., 2002). Also as a preliminary step, we compared the ASD and control groups on long-range default network connectivity, using the anterior masks in small volume corrections (Inline Supplementary Table S2).

The connectivity images were then entered into second-level analyses in SPM8 to test hypotheses at a group level. As a preliminary step, we first examined whether the ASD group and the control group exhibited default network connectivity by applying small volume corrections using masks covering the default network (posterior cingulate, precuneus, angular gyri, inferior parietal lobules, parahippocampal gyri, superior frontal gyri, anterior cingulate, BA 32, BA 10), from the Wake Forest Pickatlas (Maldjian et al., 2002). Also as a preliminary step, we compared the ASD and control groups on long-range default network connectivity, using the anterior masks in small volume corrections (Inline Supplementary Table S2).

Inline Supplementary Table S2

**Table S2:** Default network connectivity for ASD and control groups. Functional connectivity in the (A) Control group, (B) ASD group, (C) ASD > Control group, (D) Controls > ASD group. The threshold was set at *p* < 0.05 uncorrected with the number of contiguous voxels set at k ≥ 10. L = left, R = right. A full list of the default network structures used can be found in the Methods.

A). Control group						
Region	Brodmann's Area	Cluster size	*t* *df = 65*	MNI Coordinates		
				x	y	z
L posterior cingulate	23	808	14.75	− 6	− 52	24
R posterior cingulate	10	853	15.22	4	− 52	24
L precuneus	31	2062	15.55	− 6	− 50	30
	39	229	10.89	− 44	− 74	38
R precuneus	31	2291	16.42	4	− 52	32
	39	206	11.11	42	− 68	34
L angular gyrus	39	390	12.20	− 50	− 64	34
L inferior parietal lobule	39	735	11.88	− 46	− 68	38
	40	74	6.42	− 48	− 50	24
R inferior parietal lobule	40	587	12.66	50	− 62	38
	13	82	8.15	46	− 50	24
L parahippocampal gyrus	−	901	5.03	− 28	− 34	− 10
R parahippocampal gyrus	30	776	4.26	10	− 46	2
L superior frontal gyrus	10	3432	9.70	− 8	58	− 8
R superior frontal gyrus	8	3159	10.82	18	30	48
L anterior cingulate	10	810	9.09	− 2	58	2
R anterior cingulate	10	981	9.75	4	58	2
L prefrontal cortex	10	1006	10.07	− 4	56	− 8
32	311	7.80	− 2	50	0	
R prefrontal cortex	10	905	10.80	8	66	8
	32	307	8.11	6	40	− 10

(B). ASD group						
Region	Brodmann's Area	Cluster size	*t* *df = 53*	MNI Coordinates		
				x	y	z
L posterior cingulate	23	817	11.14	− 2	− 44	24
R posterior cingulate	23	818	12.10	4	− 44	24
L precuneus	31	1978	11.34	− 2	− 50	30
	19	203	7.89	− 44	− 74	40
R precuneus	31	2335	13.74	12	− 48	30
	39	233	7.00	46	− 76	34
L angular gyrus	39	381	9.13	− 46	− 68	30
R angular gyrus	39	399	9.15	48	− 74	34
L inferior parietal lobule	39	1090	8.62	− 42	− 64	38
R inferior parietal lobule	39	649	7.61	44	− 72	38
	39	294	6.43	46	− 50	22
L parahippocampal gyrus	39	54	3.27	− 10	− 48	2
	−	12	2.44	− 24	− 12	− 14
	30	34	2.02	− 14	− 34	− 6
R parahippocampal gyrus	35	379	3.73	22	− 28	− 14
L superior frontal gyrus	10	3307	7.50	− 12	66	18
R superior frontal gyrus	10	2576	6.90	8	64	24
L anterior cingulate	11	682	6.07	− 2	42	− 10
R anterior cingulate	11	719	6.44	2	42	− 10
L prefrontal cortex	10	1044	7.56	− 10	66	18
	32	260	5.79	− 2	40	− 10
R prefrontal cortex	10	849	6.90	8	64	24
	32	248	6.08	2	46	− 4

(C). Controls > ASD group						
Region	Brodmann's Area	Cluster size	*t* *df = 118*	MNI Coordinates		
				x	y	z
L posterior cingulate	31	331	2.94	− 2	− 62	24
R posterior cingulate	31	408	3.77	8	− 58	24
L precuneus	31	903	3.75	− 2	− 70	28
	19	42	2.09	− 44	− 74	38
R precuneus	31	1009	4.02	12	− 56	30
	39	33	3.11	42	− 68	34
L angular gyrus	39	72	2.29	− 50	− 72	34
R angular gyrus	39	261	3.95	44	− 66	30
L inferior parietal lobule	39	51	2.17	− 46	− 72	38
R inferior parietal lobule	39	186	3.04	46	− 70	42
L parahippocampal gyrus	35	715	3.75	− 24	− 22	− 20
R parahippocampal gyrus	20	287	2.94	34	− 22	− 28
L superior frontal gyrus	11	334	3.75	− 6	58	− 24
	6	72	2.63	− 16	24	56
	10	30	2.46	− 2	62	2
	9	92	2.43	− 4	52	28
R superior frontal gyrus	11	505	4.21	6	58	− 24
	8	1040	3.36	22	36	50
L anterior cingulate	25	63	2.88	− 2	12	− 10
R anterior cingulate	25	76	2.98	4	8	− 12
	10	97	2.60	4	58	2
L prefrontal cortex	10	132	2.56	− 8	60	− 8
R prefrontal cortex	10	375	3.66	12	66	− 4
	32	13	1.98	14	46	− 4
	32	16	1.97	8	46	4

(C). ASD group>Controls						
Region	Brodmann's Area	Cluster size	*t* *df = 118*	MNI Coordinates		
				x	y	z
L posterior cingulate	30	44	2.73	− 24	− 70	6
L precuneus	7	11	1.98	− 28	− 56	54
R precuneus	7	331	3.50	14	− 58	60
L inferior parietal lobule	40	1258	3.41	− 60	− 38	28
R inferior parietal lobule	40	1412	3.81	64	− 46	22
L superior frontal gyrus	6	520	3.58	− 4	10	54
	9	94	2.74	− 38	44	36
	10	32	2.64	− 38	58	18
R superior frontal gyrus	6	217	3.15	2	10	56
	6	164	2.62	20	− 4	74
	10	19	2.33	38	58	18
	6	12	2.06	24	4	58
L anterior cingulate	32	15	1.91	− 10	26	28
L prefrontal cortex	10	136	3.48	− 46	50	14
	10	43	3.36	− 44	50	8
	32	264	3.61	− 12	10	40
R prefrontal cortex	10	53	2.96	48	50	4
	10	26	2.51	38	58	16
	32	199	2.90	12	6	42

Inline Supplementary Table S2

Inline Supplementary Table S2 can be found online at http://dx.doi.org/10.1016/j.nicl.2012.10.008.

To address our first hypothesis, a voxel-wise multiple regression was created to examine the interaction of genotype (low expressing (S/S, S/L_G_, L_G_/L_G_) versus high expressing (L_A_/L_A_, S/L_A_, L_A_/L_G_)) by diagnosis (ASD versus control group). For this model, three regressors were entered – genotype, diagnosis, and the interaction of genotype-by-diagnosis – predicting connectivity with the posterior hub. To determine whether the beta for the interaction was significant in the anterior default network, a small volume correction was performed on the image mapping the betas of the interaction using a mask of the bilateral BA 10, where alterations in long-range default network connectivity have consistently been found in ASD samples (e.g., Monk et al., 2009; Weng et al., 2010; Wiggins et al., 2011), as well as where effects of 5-HTTLPR have been found (Wiggins et al., 2012). The small volume correction takes into account the geometric qualities of the mask when doing a correction for multiple comparisons based on the number of resels (a measure related to the number of independent observations) within the mask (Worsley et al., 1996). Significance thresholds within BA 10 were corrected for multiple comparisons using family wise error (FWE) correction (Worsley et al., 1996). Post-hoc comparisons were also performed in SPSS, comparing each subgroup pair on connectivity values extracted and averaged from a 4 mm sphere around the peak of the interaction, with a Bonferroni-corrected α level of 0.05/6 = 0.0083.

To address our second hypothesis, we created a model to examine the three-way interaction among genotype (low versus high expressing), diagnosis (ASD versus control group), and age. In this model, the three-way interaction term was entered, as well as all lower order terms. A small volume correction applied the same mask as in the first hypothesis, bilateral BA 10, to the image mapping the betas for the 3-way interaction to examine whether the three-way interaction significantly predicted connectivity with the posterior hub in the anterior default network. Post-hoc analyses were also performed to further characterize the interaction. Connectivity values from a 4 mm sphere around the peak of the 3-way interaction were extracted and averaged, then exported to SPSS. In SPSS, the simple slopes (changes in connectivity for every unit increase in age) for four subgroups (controls with low expressing genotypes, controls with high expressing genotypes, individuals with ASD and low expressing genotypes, individuals with ASD and high expressing genotypes) were tested against zero.

## 3 Results

Individuals with the low and high expressing genotypes within the control and the ASD groups did not differ on any of the symptom measures (Inline Supplementary Table S1). Both the ASD group and the control group exhibited default network connectivity, and previous findings of weaker posterior–anterior default network connectivity in the ASD group were replicated (Inline Supplementary Table S2; Cherkassky et al., 2006; Kennedy and Courchesne, 2008; Monk et al., 2009; Weng et al., 2010; Wiggins et al., 2011).

Individuals with the low and high expressing genotypes within the control and the ASD groups did not differ on any of the symptom measures (Inline Supplementary Table S1). Both the ASD group and the control group exhibited default network connectivity, and previous findings of weaker posterior–anterior default network connectivity in the ASD group were replicated ( Inline Supplementary Table S2; Cherkassky et al., 2006; Kennedy and Courchesne, 2008; Monk et al., 2009; Weng et al., 2010; Wiggins et al., 2011).

The four groups (individuals with ASD and low expressing genotypes, individuals with ASD and high expressing genotypes, controls with low expressing genotypes, controls with high expressing genotypes) did not differ in mean head motion (genotype-by-diagnosis: F_1,116_ = 0.040, *p* = .841). Additionally, age did not relate to head motion differently across the four groups (genotype-by-diagnosis-by-age: β = .058, *t*_112_ = 0.256, *p* = 0.799).

**Fig. S2:**
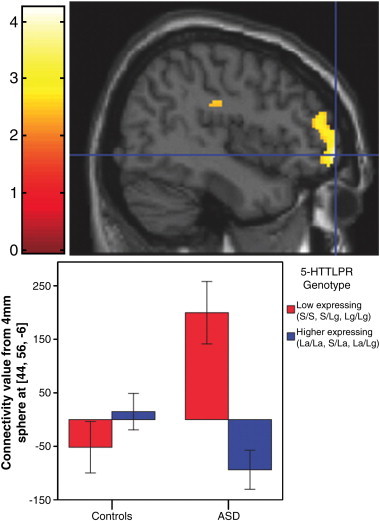
Right hemisphere — Impact of 5-HTTLPR on posterior–anterior default network connectivity differs in youth with ASD compared to controls. Voxels in color indicate places where genotype influenced connectivity between that area and the posterior hub differently for the ASD group and the control group. A significant genotype-by-diagnosis interaction in the anterior default network (xyz = 44, 56, − 6, *t*_116_ = 4.17, *p* = 0.027, corrected for multiple comparisons within bilateral BA 10) is depicted in the sagittal section of the brain (upper), with the threshold set at *p* < 0.01 for illustration purposes. To depict the interaction, contrast values from a 4 mm sphere around the peak voxel (xyz = 44, 56, − 6) were extracted and plotted (lower).

**Fig. 1:**
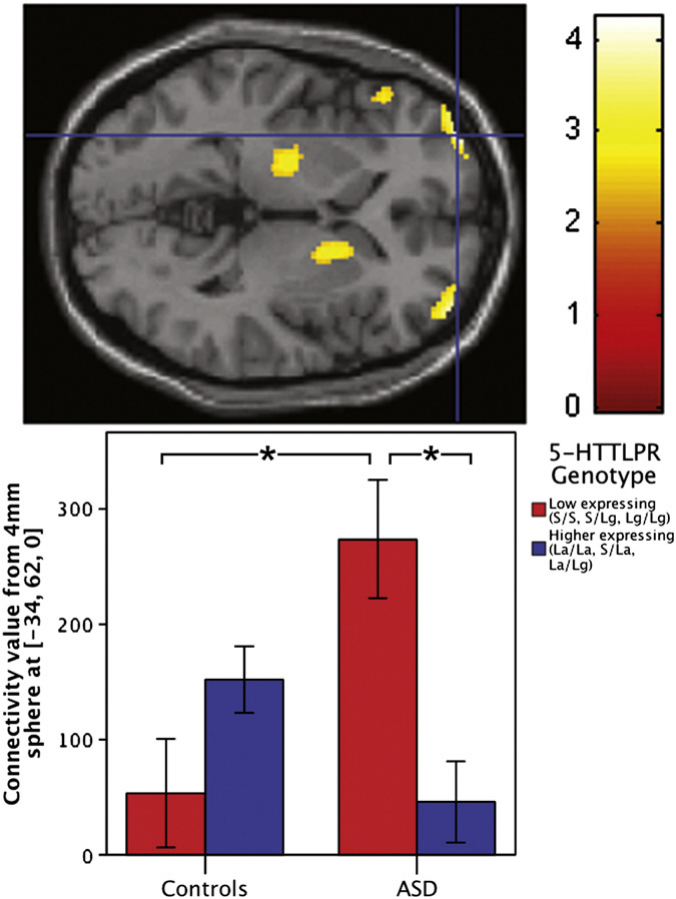
Impact of 5-HTTLPR genotypes on posterior–anterior default network connectivity is different in youth with ASD compared to controls. Voxels in color indicate places where connectivity between that area and the posterior default network is differentially influenced by 5-HTTLPR in the ASD group versus controls. A significant genotype-by-diagnosis interaction in the anterior default network (xyz = − 34, 62, 0, *t*_116_ = 4.24, *p* = 0.021, corrected for multiple comparisons within bilateral BA 10) is depicted in the transverse section of the brain (upper). For this and the subsequent brain image, the threshold was set at *p* < 0.01 for illustration purposes. To show the interaction, contrast values from a 4 mm sphere around the peak voxel (xyz = − 34, 62, 0) were extracted and plotted (lower). In the bar graph, controls show the pattern found in previous research (Wiggins et al., 2012), but youth with ASD show a different pattern. Brackets with asterisks indicate significant differences at a Bonferroni-corrected α-level of 0.05/6 = 0.0083.

The first hypothesis, that the relationship between 5-HTTLPR genotype and posterior–anterior default network connectivity strength differs in the ASD group versus controls, was confirmed. There was a significant genotype-by-diagnosis interaction predicting degree of connectivity between the posterior hub and the anterior default network in the left hemisphere (xyz = − 34, 62, 0, *t*_116_ = 4.24, *p* = 0.021, corrected for multiple comparisons within bilateral BA 10; Fig. 1). Specifically, 5-HTTLPR genotype influences posterior–anterior connectivity strength differently for individuals with ASD versus controls. Two pair-wise comparisons survived a Bonferroni correction, indicating that individuals with low expressing genotypes within the ASD group had significantly stronger connectivity than individuals with ASD and high expressing genotypes (*p* = 0.001) as well as controls with low expressing genotypes (*p* = 0.003). The genotype-by-diagnosis interaction was also significant in the right anterior default network (xyz = 44, 56, − 6, *t*_116_ = 4.17, *p* = 0.027, corrected for multiple comparisons within bilateral BA 10; Inline Supplementary Fig. S2).

The first hypothesis, that the relationship between 5-HTTLPR genotype and posterior–anterior default network connectivity strength differs in the ASD group versus controls, was confirmed. There was a significant genotype-by-diagnosis interaction predicting degree of connectivity between the posterior hub and the anterior default network in the left hemisphere (xyz = − 34, 62, 0, *t*_116_ = 4.24, *p* = 0.021, corrected for multiple comparisons within bilateral BA 10; Fig. 1). Specifically, 5-HTTLPR genotype influences posterior–anterior connectivity strength differently for individuals with ASD versus controls. Two pair-wise comparisons survived a Bonferroni correction, indicating that individuals with low expressing genotypes within the ASD group had significantly stronger connectivity than individuals with ASD and high expressing genotypes (*p* = 0.001) as well as controls with low expressing genotypes (*p* = 0.003). The genotype-by-diagnosis interaction was also significant in the right anterior default network (xyz = 44, 56, − 6, *t*_116_ = 4.17, *p* = 0.027, corrected for multiple comparisons within bilateral BA 10; Inline Supplementary Fig. S2).

Inline Supplementary Fig. S2

Inline Supplementary Fig. S2 can be found online at http://dx.doi.org/10.1016/j.nicl.2012.10.008.

Inline Supplementary Fig. S2 can be found online at http://dx.doi.org/10.1016/j.nicl.2012.10.008.

**Fig. 2:**
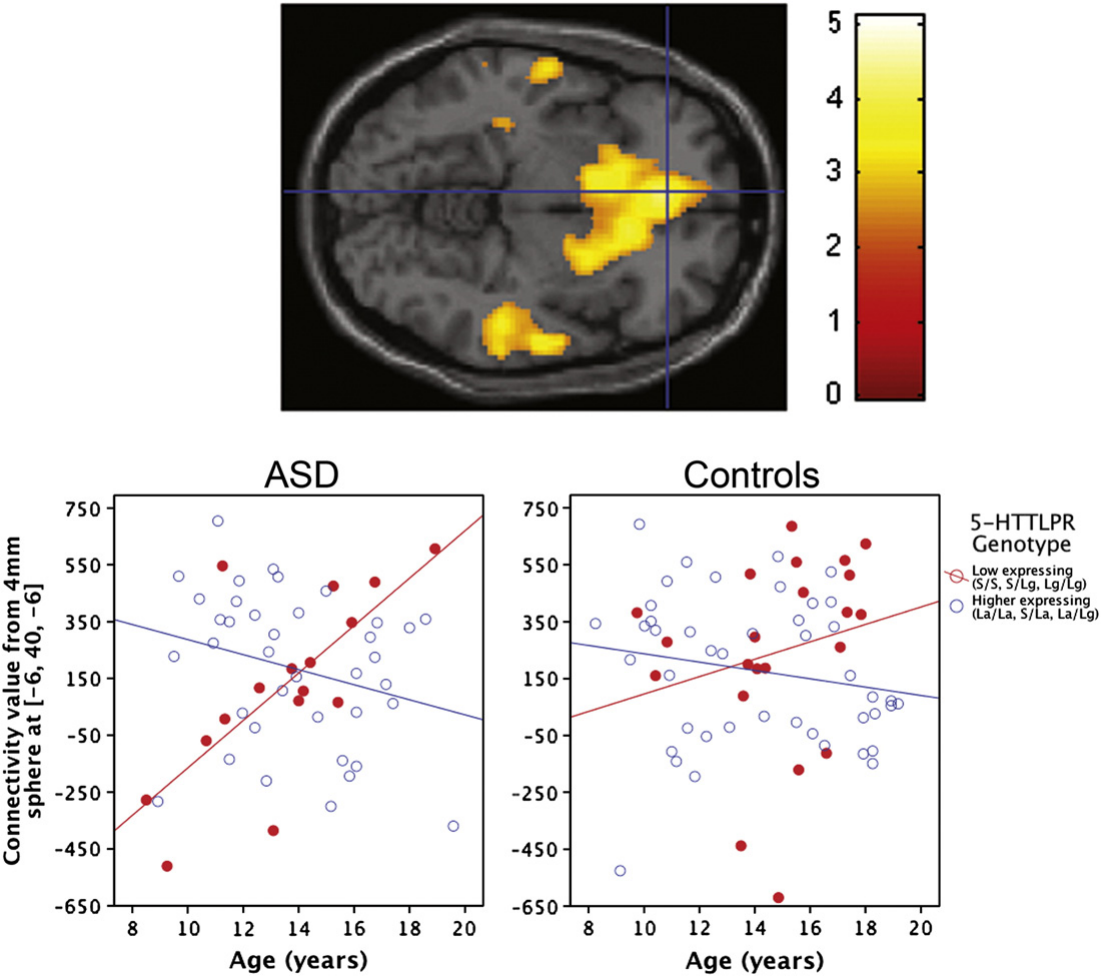
5-HTTLPR influences age-related changes in posterior–anterior default network connectivity differently in youth with ASD compared to controls. Voxels in color indicate places where connectivity between that area and the posterior hub changes across age differently for the ASD group and the control group. A significant genotype-by-diagnosis-by-age interaction in the anterior default network (xyz = − 6, 40, − 6, *t*_112_ = 4.09, *p* = 0.037, corrected for multiple comparisons within bilateral BA 10) is depicted in the transverse section of the brain (upper). To illustrate connectivity levels in each individual, contrast values from a 4 mm sphere around the peak voxel (xyz = − 6, 40, − 6) were extracted and plotted (lower).

Our second hypothesis, that the relationship between 5-HTTLPR and changes in posterior–anterior connectivity across childhood and adolescence differs in ASD compared to controls, was also confirmed. We found a significant genotype-by-diagnosis-by-age interaction predicting degree of connectivity between the posterior and anterior default network (xyz = − 6, 40, − 6, *t*_112_ = 4.09, *p* = 0.037, corrected for multiple comparisons within bilateral BA 10; Fig. 2). 5-HTTLPR genotype differentially influences age-related changes in posterior–anterior connectivity strength in individuals with ASD compared to controls. Post-hoc analyses to further characterize the interaction indicated that only individuals with ASD with low expressing genotypes had significant increases in connectivity values with age (simple slope = 0.708, *p* = 0.002), whereas the other subgroups' relationships between connectivity and age did not significantly differ from zero: ASD group, high expressing genotype, simple slope = − .268, *p* = 0.104; controls, low expressing, simple slope = 0.216, *p* = 0.335; controls, high expressing, simple slope = − .185, *p* = 0.229.

### 3.1 Additional analyses

In imaging and genetic studies with disordered populations, head motion, population stratification, psychotropic medication status, allele grouping, and degree of smoothing are potential factors influencing associations. Because of this, additional analyses were performed to determine whether these factors account for our results. As these additional analyses required a reduced sample size and/or more complex models, thereby diminishing the power to detect effects, we utilized a threshold of *p* < 0.05 without family-wise error correction. To summarize, the hypotheses were still confirmed even when taking into account each of these factors.

#### 3.1.1 Matched head motion distributions

**Fig. S3:**
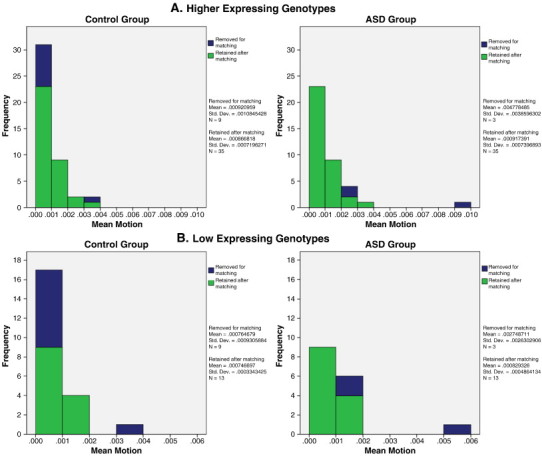
Matching participants on mean motion. Participants were randomly removed from .001 mm bins until the same number of participants remained in corresponding bins across the ASD and control groups for both (A) high expressing and (B) low expressing genotypes. Bar graph shows proportion removed in each bin.

Our matching procedure is described in Section 2.4.2 Addressing head motion. In total, 24 participants (20%) were removed in order to match the groups' motion distributions. (See Inline Supplementary Fig. S3 for a visual representation of the participants removed from each bin.) After removing participants until the group distributions were matched on mean head motion, our first hypothesis was still confirmed. The interaction of genotype-by-diagnosis significantly predicted connectivity in bilateral BA 10 (left: xyz = − 34, 62, 0, *t*_92_ = 4.22, *p* = 0.000029; right: xyz = 38, 60, 2, *t*_92_ = 3.84, *p* = 0.00011) with a subsample of participants matched on head motion. Our second hypothesis was confirmed as well with the matched subsample, as a genotype-by-diagnosis-by-age interaction significantly predicted connectivity in BA 10 (xyz = − 10, 36, − 6, *t*_88_ = 4.22, *p* = 0.000029).

Our matching procedure is described in Section 2.4.2 Addressing head motion. In total, 24 participants (20%) were removed in order to match the groups' motion distributions. (See Inline Supplementary Fig. S3 for a visual representation of the participants removed from each bin.) After removing participants until the group distributions were matched on mean head motion, our first hypothesis was still confirmed. The interaction of genotype-by-diagnosis significantly predicted connectivity in bilateral BA 10 (left: xyz = − 34, 62, 0, *t*_92_ = 4.22, *p* = 0.000029; right: xyz = 38, 60, 2, *t*_92_ = 3.84, *p* = 0.00011) with a subsample of participants matched on head motion. Our second hypothesis was confirmed as well with the matched subsample, as a genotype-by-diagnosis-by-age interaction significantly predicted connectivity in BA 10 (xyz = − 10, 36, − 6, *t*_88_ = 4.22, *p* = 0.000029).

Inline Supplementary Fig. S3

Inline Supplementary Fig. S3 can be found online at http://dx.doi.org/10.1016/j.nicl.2012.10.008.

Inline Supplementary Fig. S3 can be found online at http://dx.doi.org/10.1016/j.nicl.2012.10.008.

#### 3.1.2 Population stratification

To determine whether the findings were primarily driven by differing ancestries within our sample, 5 non-Caucasian individuals with ASD and sixteen non-Caucasian controls were excluded and the group-level analyses addressing our hypotheses were repeated. In line with our first hypothesis, including Caucasian participants only, the genotype-by-diagnosis interaction predicting connectivity strength was significant in both left (xyz = − 34, 62, − 2, *t*_95_ = 3.95, *p* = 0.000075) and right (xyz = 38, 58, 4, *t*_95_ = 3.14, *p* = 0.0011) BA 10. Supporting our second hypothesis, the genotype-by-diagnosis-by-age interaction predicting connectivity was significant in BA 10 when including only Caucasian participants (xyz = − 20, 50, 2, *t*_91_ = 3.51, *p* = 0.00035).

#### 3.1.3 Medication effects

Next, twenty-four individuals with ASD taking psychotropic medication and 1 control taking levothyroxine (a thyroid medication) were excluded before repeating the analyses. Like the analyses only including Caucasians, findings including only non-medicated participants also mirrored the original findings with the entire dataset. Supporting the first hypothesis, with only non-medicated participants, the genotype-by-diagnosis interaction was significant (left BA 10: xyz = − 34, 62, 0, *t*_91_ = 3.71, *p* = 0.00018; right BA 10: xyz = 32, 60, 6, *t*_91_ = 4.55, *p* = 0.0000084). The second hypothesis, genotype-by-diagnosis-by-age interaction predicting connectivity, also held with non-medicated participants only in BA 10 (xyz = − 6, 40, − 6, *t*_87_ = 2.97, *p* = 0.0019).

#### 3.1.4 Alternative genotype groupings

Moreover, additional analyses were conducted with alternate genotype groupings to examine whether the findings persisted when participants split into different genotype groups. First, we ran the analyses with the genotype in three groups based on expressing level: low expressing (S/S, S/L_G_, L_G_/L_G_) versus medium expressing (S/L_A_, L_A_/L_G_) vs high expressing (L_A_/L_A_) genotypes. Consistent with the first hypothesis, there was a significant genotype-by-diagnosis interaction in both the left (xyz = − 34, 62, 0, F_2,114_ = 9.22, *p* = 0.00019) and right (xyz = 44, 56, − 6, F_2,114_ = 9.19, *p* = 0.00020) anterior default network. The three-way genotype-by-diagnosis-by-age interaction was also significant with this genotype grouping in the anterior default network (xyz = − 6, 40, − 8, F_2,108_ = 11.23, *p* = 0.000037), consistent with the second hypothesis.

Second, we examined the hypotheses with the genotype grouping S/S versus heterozygotes (S/L_A_ and S/L_G_) versus L_A_/L_A_. With this alternative genotype grouping, there was a significant genotype-by-diagnosis interaction in the left (xyz = − 34, 62, − 2, F_2,114_ = 7.68, *p* = 0.00074) and right (xyz = 44, 56, − 6, F_2,114_ = 10.45, *p* = 0.000068) anterior default network. Moreover, consistent with the second hypothesis, the three-way interaction was significant in the anterior default network (xyz = − 6, 40, − 8, F_2,108_ = 8.11, *p* = 0.00052) with this alternative grouping. To summarize, the original result pattern persisted even when genotypes were grouped two alternate ways in the statistical analyses.

#### 3.1.5 5 mm smoothing kernel

The degree of smoothing can also affect results. We re-did the analyses with a 5 mm (instead of 8 mm) FWHM Gaussian kernel for spatial smoothing of the functional images. Consistent with the first hypothesis, the genotype-by-diagnosis interaction was significant in the left (xyz = − 48, 54, 6, *t*_116_ = 2.25, *p* = 0.013) and right (xyz = 42, 58, 10, *t*_116_ = 3.96, *p* = 0.00065) BA 10. Moreover, consistent with the second hypothesis, the three-way interaction was significant in BA 10 (xyz = 42, 48, 24, *t*_112_ = 3.03, *p* = 0.002) with the 5 mm smoothing kernel, albeit in a more lateral location within BA 10.

## 4 Discussion

In results confirming both the first hypothesis (genotype-by-diagnosis) and the second hypothesis (genotype-by-diagnosis-by-age), individuals with ASD and low expressing genotypes stood out among the other subgroups, exhibiting the greatest connectivity as well as the sharpest increase in connectivity values with age. This overall pattern suggests that individuals with low expressing genotypes may represent a subtype of ASD, which is consistent with previous research linking 5-HTTLPR to symptom subtypes rather than a global ASD diagnosis in a larger sample (Tordjman et al., 2001; Brune et al., 2006). Linking genotype to brain phenotypes may be a more sensitive way to identify subtypes of ASD than linking genotype to behavior, as individuals with ASD and the low and high expressing genotypes did not differ on any of the symptom measures (Inline Supplementary Table S2). Future research could examine other aspects of this potential subtype of ASD, including responsiveness to specific interventions and long-term prognosis.

In results confirming both the first hypothesis (genotype-by-diagnosis) and the second hypothesis (genotype-by-diagnosis-by-age), individuals with ASD and low expressing genotypes stood out among the other subgroups, exhibiting the greatest connectivity as well as the sharpest increase in connectivity values with age. This overall pattern suggests that individuals with low expressing genotypes may represent a subtype of ASD, which is consistent with previous research linking 5-HTTLPR to symptom subtypes rather than a global ASD diagnosis in a larger sample (Tordjman et al., 2001; Brune et al., 2006). Linking genotype to brain phenotypes may be a more sensitive way to identify subtypes of ASD than linking genotype to behavior, as individuals with ASD and the low and high expressing genotypes did not differ on any of the symptom measures (Inline Supplementary Table S2). Future research could examine other aspects of this potential subtype of ASD, including responsiveness to specific interventions and long-term prognosis.

There are two main possibilities to explain why 5-HTTLPR influences the ASD group and control group differently. First, a gene-by-gene interaction may account for our results. The specifics of the complex genetic etiology of ASD are a subject of intense inquiry. Nonetheless, as ASD is highly heritable (Miles, 2011), individuals with ASD may carry a systematically different overall genetic profile than controls. Causative gene products may interact with 5-HTTLPR, leading to alterations in expression levels that then produce a different brain phenotype. Future research probing this possibility may include examining other autism genes and their involvement in serotonin metabolism.

Alternatively, a gene-by-environment interaction may explain our findings. As 5-HTTLPR is sensitive to environmental input (Belsky et al., 2009), it may be that 5-HTTLPR affects brain function in individuals with ASD differently than controls because individuals with ASD experience an altered social environment brought about by the reactions of others to their symptoms. Particularly during adolescence, an important social development period in which relationships with peers become more important (Youniss and Haynie, 1992), individuals with ASD may miss out on social opportunities with peers and thus find themselves in an environment with reduced social stimuli. This environment could affect epigenetically-sensitive serotonin transporter expression and subsequently, brain function. Future studies incorporating comprehensive environmental measures and focusing on molecular mechanisms of altered serotonin transporter expression, such as methylation, will be necessary to probe this possibility.

This study has several limitations, as some confounds make imaging and genetics research, particularly with pediatric clinical populations, more challenging. First, in our study, mean head motion did not differ among the groups we compared: individuals with ASD and low expressing genotypes, individuals with ASD and high expressing genotypes, controls with low expressing genotypes, and controls with high expressing genotypes. Also, age did not relate to head motion differently across the four groups. Nevertheless, motion remains a concern in all functional connectivity studies, so we took several steps to address motion: first, only participants with movement under 2.5 mm and 2.5° in all translation and rotation directions were included; second, we realigned the functional images; third, we removed variance associated with movement in the x, y, z, roll, pitch, and yaw directions; fourth, we repeated the analyses with a subsample matched on head motion and found that our hypotheses were still confirmed even when motion distributions were the same across individuals with ASD and controls in the low and high expressing genotype groups.

Another limitation is that the cross-sectional design utilized in this study precludes inferences about developmental trajectories within individuals. Future studies may use a longitudinal design to rule out birth cohort effects. Additionally, longitudinal studies will be useful to examine whether brain differences earlier in development predict later symptom presentation and responsiveness to particular treatments.

Third, we did not exclude any racial or ethnic group when recruiting participants, which can contribute to spurious associations in genetic studies. Although it should be acknowledged that Caucasians in our sample may not all be of the same ancestry, we repeated the analyses with non-Caucasian participants removed to determine whether results were primarily due to several different ancestries within the sample. The genotype-by-diagnosis and genotype-by-diagnosis-by-age interactions predicting posterior–anterior connectivity were found even with non-Caucasians excluded from the analyses, suggesting that the results were not primarily driven by population stratification. Nevertheless, the lack of understanding of genetic effects in different racial/ethnic groups is a widespread problem in the field that must be addressed in future work.

### 4.1 Conclusions

This is the first study, to our knowledge, to examine the influence of 5-HTTLPR genotype on the default network in individuals with ASD. We found that the relationship between 5-HTTLPR genotype and posterior–anterior default network connectivity is different in individuals with ASD compared to controls. Specifically, consistent with previous research in controls (Wiggins et al., 2012), high expressing genotypes were associated with stronger connectivity than low expressing genotypes. However, the pattern was reversed for the ASD group: individuals with ASD and low expressing genotypes had stronger connectivity than individuals with ASD and high expressing genotypes. Also, we found that youth with ASD and low expressing genotypes had greater age-related increases in connectivity values compared to others in the ASD group with high expressing genotypes and to controls with either low or high expressing genotypes. The present findings provide evidence that the cascade of events from genetic variation to brain function is markedly different in ASD versus typically developing, healthy individuals. Moreover, the findings suggest that the impact of genotype on brain function is not static but rather develops and changes with age. Thus, understanding how 5-HTTLPR affects brain function in ASD is dependent on the developmental timeframe.

Although replication of our findings with a larger sample is necessary, the present study lays the groundwork to better understand the genetic and brain mechanisms that are involved in ASD. The present study documented a different impact of 5-HTTLPR on both default network connectivity and the development of default network connectivity in ASD compared to controls. Future studies may expand on these findings by examining the structural connections within the default network in vivo using diffusion tensor imaging. Moreover, the resting connectivity approach used in this study will be useful to examine the brain activation patterns of lower functioning individuals with ASD or very young children. These individuals are underrepresented in functional MRI studies because they are often unable to comply with the demands of a task requiring responses in the scanner. The relatively low demand of a resting fMRI acquisition, on the other hand, may allow lower functioning and younger participants to be successfully scanned. Obtaining brain data from individuals with a greater range of cognitive abilities and ages will allow researchers to gain a broader, more representative picture of ASD and the developmental trajectory of ASD earlier than mid-childhood. To conclude, the findings from our study open a path for a research program to better understand genetic influences on brain function in ASD.

## Conflict of interest

C.L. receives royalties from the publisher of diagnostic instruments used on participants in this paper, Western Psychological Services. She gives all profits to charity.
