# Supplementary material for: The impact of serotonin transporter genotype on default network connectivity in children and adolescents with autism spectrum disorders
Source: Neuroimage Clin. 2012 Nov 7;2:17–24. doi: 10.1016/j.nicl.2012.10.008 (PMC3777679; doi:10.1016/j.nicl.2012.10.008)
Supplement: Inline Supplementary Table S2 [file mmc2.pdf]

Supplementary Table 2. Default network connectivity for ASD and control groups. Functional connectivity in the (A) Control group, (B) ASD group, (C) ASD > Control group, (D) Controls > ASD group. The threshold was set at $p < 0.05$ uncorrected with the number of contiguous voxels set at k ≥ 10. L = left, R = right. A full list of the default network structures used can be found in the Materials and methods section.

(A). Control group

Region	Brodmann's Area	Cluster size	*t* *df = 65*	MNI Coordinates x	y	z
L posterior cingulate	23	808	14.75	-6	-52	24
R posterior cingulate	10	853	15.22	4	-52	24
L precuneus	31	2062	15.55	-6	-50	30
	39	229	10.89	-44	-74	38
R precuneus	31	2291	16.42	4	-52	32
	39	206	11.11	42	-68	34
L angular gyrus	39	390	12.20	-50	-64	34
R angular gyrus	39	396	13.89	50	-62	30
L inferior parietal lobule	39	735	11.88	-46	-68	38
	40	74	6.42	-48	-50	24
R inferior parietal lobule	40	587	12.66	50	-62	38
	13	82	8.15	46	-50	24
L parahippocampal gyrus	--	901	5.03	-28	-34	-10
R parahippocampal gyrus	30	776	4.26	10	-46	2
L superior frontal gyrus	10	3432	9.70	-8	58	-8
R superior frontal gyrus	8	3159	10.82	18	30	48
L anterior cingulate	10	810	9.09	-2	58	2
R anterior cingulate	10	981	9.75	4	58	2
L prefrontal cortex	10	1006	10.07	-4	56	-8

	32	311	7.80	-2	50	0
R prefrontal cortex	10	905	10.80	8	66	8
	32	307	8.11	6	40	-10

(B). ASD group

**Region**	**Brodmann's Area**	**Cluster size**	***t*** ***df = 53***	**MNI Coordinates** x	y	z
L posterior cingulate	23	817	11.14	-2	-44	24
R posterior cingulate	23	818	12.10	4	-44	24
L precuneus	31	1978	11.34	-2	-50	30
	19	203	7.89	-44	-74	40
R precuneus	31	2335	13.74	12	-48	30
	39	233	7.00	46	-76	34
L angular gyrus	39	381	9.13	-46	-68	30
R angular gyrus	39	399	9.15	48	-74	34
L inferior parietal lobule	39	1090	8.62	-42	-64	38
R inferior parietal lobule	39	649	7.61	44	-72	38
	39	294	6.43	46	-50	22
L parahippocampal gyrus	39	54	3.27	-10	-48	2
	--	12	2.44	-24	-12	-14
	30	34	2.02	-14	-34	-6
R parahippocampal gyrus	35	379	3.73	22	-28	-14
L superior frontal gyrus	10	3307	7.50	-12	66	18
R superior frontal gyrus	10	2576	6.90	8	64	24
L anterior cingulate	11	682	6.07	-2	42	-10
R anterior cingulate	11	719	6.44	2	42	-10
L prefrontal cortex	10	1044	7.56	-10	66	18
	32	260	5.79	-2	40	-10

R prefrontal cortex	10	849	6.90	8	64	24
	32	248	6.08	2	46	-4

(C). Controls > ASD group

**Region**	**Brodmann's Area**	**Cluster size**	***t*** ***df = 118***	**MNI Coordinates** **x**	**y**	**z**
L posterior cingulate	31	331	2.94	-2	-62	24
R posterior cingulate	31	408	3.77	8	-58	24
L precuneus	31	903	3.75	-2	-70	28
	19	42	2.09	-44	-74	38
R precuneus	31	1009	4.02	12	-56	30
	39	33	3.11	42	-68	34
L angular gyrus	39	72	2.29	-50	-72	34
R angular gyrus	39	261	3.95	44	-66	30
L inferior parietal lobule	39	51	2.17	-46	-72	38
R inferior parietal lobule	39	186	3.04	46	-70	42
L parahippocampal gyrus	35	715	3.75	-24	-22	-20
R parahippocampal gyrus	20	287	2.94	34	-22	-28
L superior frontal gyrus	11	334	3.75	-6	58	-24
	6	72	2.63	-16	24	56
	10	30	2.46	-2	62	2
	9	92	2.43	-4	52	28
R superior frontal gyrus	11	505	4.21	6	58	-24
	8	1040	3.36	22	36	50
L anterior cingulate	25	63	2.88	-2	12	-10
R anterior cingulate	25	76	2.98	4	8	-12
	10	97	2.60	4	58	2

L prefrontal cortex	10	132	2.56	-8	60	-8
R prefrontal cortex	10	375	3.66	12	66	-4
	32	13	1.98	14	46	-4
	32	16	1.97	8	46	4

(C). ASD > Control group

**Region**	**Brodmann's Area**	**Cluster size**	***t*** ***df = 118***	**MNI Coordinates** **x**	**y**	**z**
L posterior cingulate	30	44	2.73	-24	-70	6
L precuneus	7	11	1.98	-28	-56	54
R precuneus	7	331	3.50	14	-58	60
L inferior parietal lobule	40	1258	3.41	-60	-38	28
R inferior parietal lobule	40	1412	3.81	64	-46	22
L superior frontal gyrus	6	520	3.58	-4	10	54
	9	94	2.74	-38	44	36
	10	32	2.64	-38	58	18
R superior frontal gyrus	6	217	3.15	2	10	56
	6	164	2.62	20	-4	74
	10	19	2.33	38	58	18
	6	12	2.06	24	4	58
L anterior cingulate	32	15	1.91	-10	26	28
L prefrontal cortex	10	136	3.48	-46	50	14
	10	43	3.36	-44	50	8
	32	264	3.61	-12	10	40
R prefrontal cortex	10	53	2.96	48	50	4
	10	26	2.51	38	58	16
	32	199	2.90	12	6	42
